# Cardiovascular risk stratification in familial hypercholesterolaemia

**DOI:** 10.1136/heartjnl-2015-308845

**Published:** 2016-04-28

**Authors:** Mahtab Sharifi, Roby D Rakhit, Steve E Humphries, Devaki Nair

**Affiliations:** 1 Department of Clinical Biochemistry, Royal Free Hospital, London, UK; 2 Department of Cardiology, Royal Free Hospital and Institute of Cardiovascular Science, University College London, London, UK; 3 Cardiovascular Genetic Centre, University College London, London, UK

## Abstract

Familial hypercholesterolaemia (FH) is a common autosomal-dominant disorder in most European countries. Patients with FH are characterised by a raised level of low-density lipoprotein cholesterol and a high risk of premature coronary heart disease (CHD). Currently there is no consensus regarding the clinical utility to predict future coronary events or testing for the presence of subclinical atherosclerotic disease in asymptomatic patients with FH. Family screening of patients with FH as recommended by the UK National Institute of Health and Care Excellence guideline would result in finding many young individuals with a diagnosis of FH who are clinically asymptomatic. The traditional CHD risk scores, that is, the Framingham score, are insufficient in risk prediction in this group of young individuals. In addition, a better understanding of the genetic aetiology of the FH phenotype and CHD risk in monogenic FH and polygenic hypercholesterolaemia is needed. Non-invasive imaging methods such as carotid intima-media thickness measurement might produce more reliable information in finding high-risk patients with FH. The potential market authorisation of novel therapeutic agents such as PCSK9 monoclonal inhibitors makes it essential to have a better screening programme to prioritise the candidates for treatment with the most severe form of FH and at higher risk of coronary events. The utility of new imaging techniques and new cardiovascular biomarkers remains to be determined in prospective trials.

## Introduction

Familial hypercholesterolaemia (FH) is a common autosomal-dominant disorder with the frequency of heterozygous FH estimated at 1 in 200–500 in most European populations.^1^ The clinical diagnosis of FH is based on a raised low-density lipoprotein cholesterol (LDL-C) level of >4.9 mmol/L, physical stigmata, for example, tendon xanthomata or evidence of these signs in first-degree or second-degree relatives and having a personal or family history of premature coronary heart disease (CHD).^2^ Mutations in three genes, the LDL-receptor gene (*LDLR*), the gene coding for apolipoprotein B (*APOB*) and the gene encoding the proprotein convertase subtilisin/kexin type 9, are responsible for causing FH. In practice, around 60% of the patients with a clinical diagnosis of FH do not have a detectable mutation in any of these three common genes^3^ and in such patients it has been suggested that there is most likely to be a polygenic cause for their raised LDL-C level.^4^

CHD is the biggest cause of mortality and morbidity in individuals diagnosed with FH. Data from the Simon Broome Registry showed that the cumulative risk of a fatal or non-fatal coronary event in the patients with FH by the age of 60 years without effective treatment is at least 50% in men and 30% in women with a marked increase in postmenopausal women.^5^ The coronary mortality was reported to be fourfold higher in people with a clinical diagnosis of definite FH compared with the general population.^6^

The 2008 National Institute of Health and Care Excellence (NICE) guideline on FH recommends cascade screening for first-degree relatives of all patients with a clinical diagnosis of FH.^2^ This would result in finding many individuals with a diagnosis of FH who are clinically asymptomatic. Risk stratification of subclinical coronary atherosclerosis in asymptomatic patients with FH who are at higher risk of advanced CHD is important, as these people might be eligible for intensive treatment at a younger age. There is currently no reliable screening test available to predict prevalence and progression of atherosclerosis and major adverse cardiovascular events in asymptomatic individuals with FH. The purpose of this paper is to review the current evidence on cardiovascular risk stratification in this group.

## CHD risk in heterozygous FH

Premature CHD is an established phenomenon of FH, with the average mean age of onset of coronary symptoms shown to be 45 years in men and 55 years in women.^5^ Because the physical stigmata of FH develop later in life, establishing a diagnosis in young individuals is often difficult. Since the age of onset and the severity of the CHD in patients with FH vary, it is difficult to decide how aggressive the treatment to prevent the progress of atherosclerosis should be and how to monitor the CHD progression in these patients. The current FH guidelines recommend that existing risk algorithms such as Framingham risk score should not be used in patients with FH.^2^ ^7^ These risk scores are based on data from general population and they significantly underestimate the lifetime CHD risk in patients with FH who have grossly elevated LDL-C levels since birth.

### Polygenic versus monogenic FH

There are several lines of evidence to suggest that the extent of atherosclerosis is likely to be higher in monogenic compared with polygenic patients. The Simon Broome Registry showed that patients with a clinical diagnosis of definite FH had a higher standardised mortality ratio for CHD than those with a clinical diagnosis of possible FH (2.94 vs 2.05).^8^ Since a mutation can be found in ∼80% of patients with definite FH, the majority of this group have a monogenic cause. By contrast, a mutation can only be detected in 25%–30% of patients with possible FH,^9^ meaning the majority of these ‘lower risk’ patients with FH have a polygenic cause for their elevated LDL-C level.

A second body of evidence comes from the Simon Broome DNA study where the prevalence of CHD in patients with the three different genetic causes of FH was examined. After adjustment for age, sex, smoking and blood pressure, compared with the patients where no mutation had been identified, the OR for having CHD in patients with the *LDLR*, *APOB* or *PCSK9* mutations were 1.84, 3.4 and 19.9, respectively (table 1), with an overall significant difference among groups (p=0.003).^10^ The no-mutation group from the Simon Broome study was included in the gene score analysis in the Talmud *et al*^4^ paper and had the expected high gene score, that is, they had polygenic hypercholesterolaemia. The implication of these data is that, compared with those with a monogenic cause of FH, the prevalence of CHD in those with a polygenic cause is *lower*.

**Table 1:** The OR for coronary heart disease (CHD) by mutation type adjusted for risk factors (table from Humphries *et al*^10^)

Mutation	Patients with CHD (n)	Patients without CHD (n)	OR (95% CI)*
None	55	101	1
*LDLR*	91	145	1.84 (1.10 to 3.06), p=0.02
*APOB*	6	4	3.40 (0.71 to 16.36), p=0.13
*PCSK9*	6	1	19.96 (1.88 to 211.55), p=0.01

*OR for having CHD adjusted for age, sex, smoking and systolic blood pressure at recruitment compared with the patients where no mutation was identified.

*APOB*, apolipoprotein B; CHD, coronary heart disease; *LDLR*, LDL-receptor gene; *PCSK9*, protein convertase subtilisin/kexin 9.

There are several possible reasons why CHD risk is higher in monogenic versus polygenic patients but is most likely to be due to the fact that the monogenic group have had severely elevated LDL-C level since birth, and thus, have a greater cumulative ‘LDL-C burden’,^7^ while the polygenic group have developed elevated LDL-C level only with increasing age. It may also be that in the polygenic group, other CHD risk factors are less prevalent, for example, higher levels of lipoprotein(a) or lower levels of high-density lipoprotein cholesterol (HDL-C), both of which were suggested as being important in determining the risk of CHD.^5^ Studies are needed to examine, for example, carotid atherosclerosis by ultrasound or coronary calcification by CT scan in these two patient groups. However, if confirmed that the level of atherosclerosis in polygenic patients is lower than in monogenic patients with FH, it would suggest that these individuals could be appropriately managed in the communities under general practitioners and could, thus, be discharged from hospital tertiary lipid clinics. This will allow the appropriate use of resources for patients with a known mutation who are at highest risk of CHD.

## Genetic and biochemical risk factors

Genetic mutations that severely impair the function of LDLR (null allele *LDLR* mutations) are associated with more advanced degree of CHD and an earlier onset. The specific gain-of-function *PCSK9* mutation (p.Asp374Tyr), a common mutation in lipoprotein lipase gene (p.Asn291Ser),^11^ *ACE* DD genotype^12^ and genetic polymorphisms, for example, presence of E2 and E4 alleles in apolipoprotein E,^13^ are known to increase the CHD risk in patients with FH, while some *PCSK9* loss-of-function variants and *APOB* gene mutations are associated with a lower risk. Several genome-wide association studies have identified common variants associated with increase in the risk of CHD in the general population; however, no genetic risk variant for CHD in individuals with FH has been identified so far and the genetics of CHD risk in FH seems more complex.^14^

Traditional risk factors such as age, male gender, smoking, hypertension, higher LDL-C level and lower HDL-C level, all play a role in patients with FH but their predictive value is different from the general population.^5^ Not all the individuals with FH develop atherosclerosis and CHD to same extent and they might even show severe accelerated atherosclerosis despite no features of metabolic syndrome. Although cardiovascular risk in patients with FH is mainly driven by the degree of elevation of LDL-C level, the risk of CHD in FH is not solely due to elevated LDL-C level and its severity and clinical expression is even variable within a family, where all relatives carry the same *LDLR* gene defect.^15^ A family history of an early coronary event in first-degree or second-degree relatives generally puts the patient at higher risk.^14^ Low HDL-C level and high total:HDL-C ratio are strongly associated with a risk of CHD in FH.^16^

Lipoprotein(a) is an established risk factor for cardiovascular disease^17^ and, irrespective of LDL-C levels, its serum level has been consistently reported to be significantly higher in patients with FH, especially in those with an early CHD event.^18^ Lipoprotein(a) measurement is recommended in all subjects at intermediate and high risk of CHD, for example, patients with FH.^17^ There is limited evidence available in using new cardiac biomarkers such as high-sensitivity C reactive protein and inflammatory cytokines in risk stratification of asymptomatic patients with FH, which have been examined only in case–control studies with small number of participants. Whether there is any benefit in adding genetic and novel biochemical biomarkers in CHD risk prediction criteria for patients with FH needs further large-scale studies.

### Imaging techniques

Non-invasive imaging modalities might be another way to identify asymptomatic individuals with higher cardiovascular risk. Imaging techniques were recommended to screen asymptomatic people at intermediate and high risk in the 2012 European Society of Cardiology Guidelines for Cardiovascular Prevention.^19^

### Carotid intima-media thickness measurement

Over the past few years, a large number of studies have reported on the association between increased carotid intima-media thickness (cIMT) and the risk of cardiovascular disease in the general population. The ‘IMPROVE’, a multicentre European study, showed that all cIMT measures (common, bifurcation and internal carotid arteries) have a value in relation to an increased risk of cardiovascular disease.^20^

The value of cIMT in cardiovascular risk stratification in the general population is an ongoing debate. Addition of cIMT measurement to traditional cardiovascular risk prediction models in the normal population does not lead to a significant increase in the performance of those models or leads to only a small improvement in the risk prediction.^21^ However, several clinical trials showed that the cIMT changes remain sensitive to the changes in the LDL-C levels and it has been consistently shown that cIMT can be used in evaluation of the carotid atherosclerosis progression.

Individuals with FH have higher carotid IMT and femoral IMT compared with people with normal lipid levels or other types of inherited hypercholesterolaemia such as familial combined hypercholesterolaemia.^22^ Also, among patients with FH, individuals carrying *LDLR* null alleles have higher cIMT measurements than those with *LDLR* defective alleles.^23^ The cIMT in patients with FH might help to differentiate patients with a severe form of FH with more advanced atherosclerosis who are at higher risk of CHD.

The cIMT has been found to be significantly higher among dyslipidaemic children compared with children with normal lipid levels.^24^ A 10-year follow-up study in statin-treated children with FH and their unaffected siblings showed that the mean cIMT was significantly greater in children with FH even after 10 years of treatment with lipid-lowering medication. However, progression of the cIMT from baseline remained similar in both groups.^25^

In addition to simple cIMT measurements, several new functional parameters derived from cIMT ultrasound images that are currently under investigation might give valuable information to identify patients with an elevated risk of future cardiovascular events. These new parameters include three-dimensional cIMT scanning to visualise vessel wall intima morphology^26^ and cIMT variability to assess surface pattern and extent of abnormality in carotid arteries.^27^

### CT scan and MRI

Cardiac CT is a useful non-invasive imaging modality to assess coronary artery atherosclerosis in symptomatic and asymptomatic high-risk patients. Coronary artery calcification has been shown to be a surrogate marker for atherosclerosis, with the calcium score ‘Agatston score’, being proportional to atherosclerosis plaque burden and cardiovascular risk.^28^ It has limitation in its diagnostic value in accurately evaluating the severity of CHD and plaque vulnerability. Direct examination of the vessel lumen using CT coronary angiography has shown a diagnostic capability comparable with that of invasive methods for visualisation of the anatomical details and degree of coronary lumen stenosis and for assessment of the plaque burden.^29^

Dyslipidaemia is associated with an increased prevalence of soft plaques. The key feature of this vulnerable subgroup of plaques is the large lipid/necrotic core covered by a thin fibrous cap. Very few studies have been done on assessing plaque composition in patients with FH. Most data come from studies of patients who underwent lipid-lowering therapies such as apheresis and reported a decrease in the lipid component of atherosclerotic plaques with aggressive treatment.

High-resolution cardiovascular magnetic resonance (CMR) has become a reliable technique to assess atherosclerotic plaque morphology, showing a good correlation with histopathology. It is non-invasive and can reliably quantify carotid atherosclerosis. Underhill *et al*^30^ carried out a double-blind trial in 43 patients with raised cholesterol levels who received rosuvastatin for 24 months. They reported a significant reduction in lipid-rich necrotic core plaque in carotid arteries measured by 1.5-T CMR, whereas the overall plaque burden remained unchanged over the course of treatment. Whether the data on carotid plaque burden will add more information to the current cardiovascular risk assessment in patients with FH is not clear.

Only a few studies have been done in asymptomatic patients with FH to report calcium score and plaque burden by CT scan or to assess aortic wall calcification and lipid-rich plaques by MRI (table 2).^31–41^

**Table 2:** Results of the studies with CT scan and MRI in asymptomatic individuals with heterozygous FH

Study	FH subjects (n)	Controls (n)	Imaging technique	Results
ten Kate *et al*^31^	67	30 healthy subjects	CTCA	Patients with FH had greater coronary calcium score.
Viladés Medel *et al*^32^	50	70 healthy subjects	CTCA	Patients with FH had a greater prevalence, extension and severity of subclinical CHD.
Ten Kate *et al*^33^	59 patients with FH with null mutation	86 patients with FH with reduced or normal LDLR function	CTCA	LDLR-negative patients had higher number of diseased coronary artery segments per patient.
Neefjes *et al*^34^	140 patients with FH with follow-up scans	–	CTCA	About 54% of all coronary plaques were calcified.
Neefjes *et al*^35^	101	126 patients without FH having non-angina chest pain	CTCA	Total calcium score was significantly higher in patients with FH.
Miname *et al*^36^	102	35 healthy subjects	CTCA	Patients with FH had a significantly higher number of plaques, stenosis, segments with plaques and calcium scores.
Martinez *et al*^37^	89	31 healthy subjects	16 or 64 sliced CT	Coronary artery calcification prevalence and severity were higher in FH.
Ye *et al*^38^	32	34 healthy subjects	Electron-beam CT	Coronary artery calcification was higher in FH.
Caballero *et al*^39^	36	19 healthy subjects	MRI of aorta	Atherosclerotic plaques in descending aorta were significantly higher in FH cases.
Soljanlahti *et al*^40^	39	25 healthy subjects	MRI of aorta	No difference in any of the morphological or functional aortic parameters between patients and controls detected.
Schmitz *et al*^41^	11	26 subjects	MRI of aorta	The descending thoracic aorta wall area was significantly larger in patients with FH.

CHD, coronary heart disease; CTCA, CT coronary angiography; FH, familial hypercholesterolaemia; LDLR, LDL-receptor gene.

### Other techniques

A number of clinical studies confirmed the association between endothelial dysfunction and increased arterial stiffness with higher risk of cardiovascular disease in general population. Endothelial dysfunction in FH occurs from early age in childhood. In a study of 60 asymptomatic patients, 21 with a confirmed FH-causing mutation, 19 with an elevated LDL-C level but no FH-causing mutation and 20 healthy controls, brachial artery flow-mediated dilation was significantly lower in all patients with a raised LDL-C level compared with the healthy group, but arterial stiffness parameters were similar.^42^ This suggests that the FH mutation by itself is not a main indicator of endothelial dysfunction and that other factors are involved.

Invasive methods are not recommended for risk stratification in asymptomatic patients. Myocardial perfusion imaging modalities, such as stress echocardiography, nuclear myocardial perfusion tests and magnetic resonance myocardial perfusion imaging, are only recommended for patients presenting with the clinical symptom of chest pain. Methods such as intravascular ultrasound and optical coherence tomography as well as the near-infrared spectroscopy (NIRS) have been used to assess plaque morphology in coronary arteries recently; however, there are not enough data available in their usage in asymptomatic patients.

## CHD risk in homozygous FH

Homozygous FH (HoFH) is a rare condition with a prevalence of one to three cases per million in most populations.^43^ It is characterised by extremely accelerated atherosclerosis that occurs in coronary arteries and in all major arteries in the body, for example, carotid arteries, thoracic aorta, renal arteries and other peripheral arteries. The first major cardiovascular event in these patients often occurs during adolescence with angina and myocardial infarction in early childhood, typically in the individuals who are *LDLR*-negative.^44^ The newer imaging techniques might be useful in HoFH cases. HoFH is typically refractory to existing lipid-lowering medications and therefore lipoprotein apheresis is recommended in patients with HoFH as soon as possible, ideally by age 5 and not later than 8 years.^45^

## Treatment and prevention

The UK NICE guideline recommends statins as the first-line treatment for patients with FH and a reduction of at least 50% in LDL-C concentration from baseline level.^2^ A decrease in serum LDL-C levels should have an influence on prevalence of soft plaques and plaque composition and may lower the fraction of vulnerable plaques among atherosclerotic lesions.

**Figure 1:**
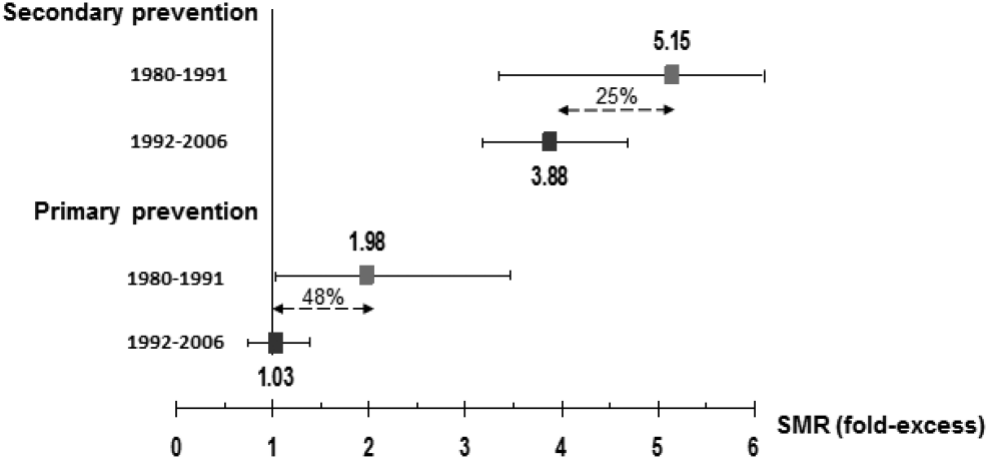
Standard mortality ratio (SMR)-fold excess for coronary heart disease (CHD) in patients with a diagnosis of familial hypercholesterolaemia (FH) with and without statin treatment (1980–1991 vs 1992–2006) is shown. (Data from Neil *et al*,^46^ 2766 definite and possible patients with FH (1456 men and1310 women) with 190 CHD deaths observed and 37 727 person-years follow-up.)

**Figure 2:**
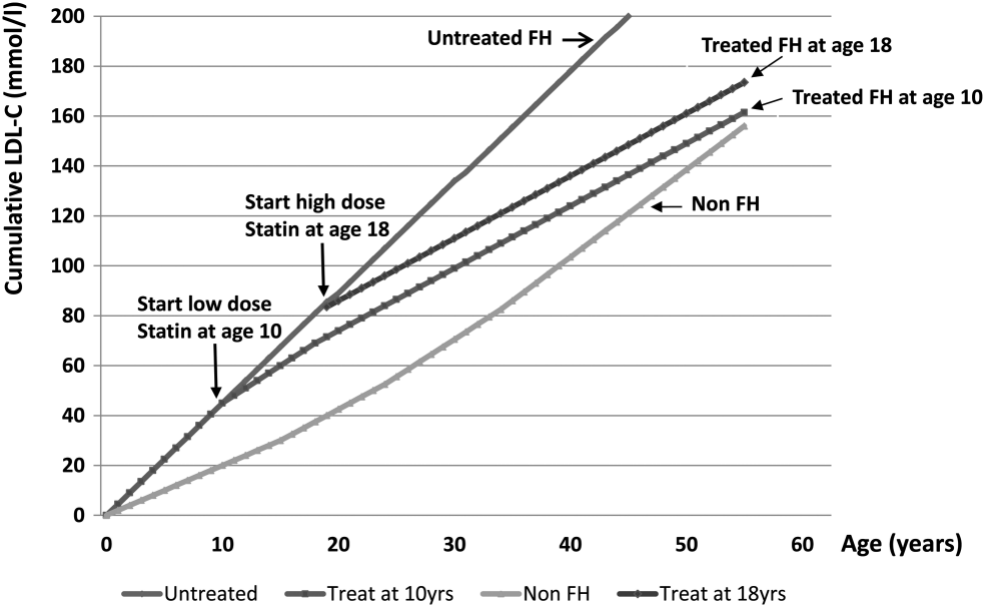
Cumulative LDL burden, expressed as mmol/L/year, over a lifetime in individuals having non-familial hypercholesterolaemia (FH) and individuals having FH with and without treatment showing threshold for coronary heart disease. (Data from Starr *et al*^47^ and Vuorio *et al*^48^.) LDL-C, low-density lipoprotein cholesterol.

In FH, primary prevention interventions are more effective in terms of absolute number of prevented deaths than interventions in the setting of secondary prevention. The major benefit of statin treatment appears to be in primary prevention of fatal coronary disease. Data from Simon Broome Registry in the UK (1980–2006) showed 48% reduction in CHD mortality, from a twofold excess to none, when statin was taken for primary prevention compared with 25% reduction when only stains were used for secondary prevention (figure 1).^46^ Based on data from Starr *et al*^47^ study, LDL burden would be delayed in early treated subjects with FH by 5 years in comparison to the subjects starting statin treatment at the age of 18 (figure 2).^48^ This suggests that with earlier diagnosis and treatment, it should be possible to prevent any excess coronary mortality in early adulthood.

For a small proportion of patients with FH with very high LDL-C level, who have progressive coronary disease despite maximal statin treatment or who are statin intolerant, the PCSK9 inhibitors may offer additional therapeutic options in future. These have been shown to effectively reduce LDL-C levels in patients with heterozygous FH and patients with HoFH who have some residual LDLR activity that can be rescued by PCSK9 blockade,^49^ but not in those with LDLR null alleles.^50^ Since patients with the *PCSK9* mutation have the highest untreated and on-treatment LDL-C levels and the highest CHD risk,^10^ this group may be appropriate for these agents. By contrast, those with a clinical diagnosis of FH but with no detectable mutation but a polygenic high LDL-C single-nucleotide polymorphism score as their genetic cause are likely to be adequately treated with more modest doses of currently available statin agents. Thus, a better understanding of the genetic aetiology of the FH phenotype in the future should enable the prioritisation of patients with the most severe form of FH, who are at higher risk of cardiovascular events, to use these new treatments.

## Conclusion

Currently, there is limited evidence available to guide the optimum cardiovascular risk stratification of patients with FH. Traditional risk factors are insufficient for risk prediction in this young group of patients and other methods such as non-invasive imaging might produce information that is more reliable.
